# Supplementary material for: Achieving single nucleotide sensitivity in direct hybridization genome imaging
Source: Nat Commun. 2022 Dec 15;13:7776. doi: 10.1038/s41467-022-35476-y (PMC9755149; doi:10.1038/s41467-022-35476-y)
Supplement: Supplementary file 1 — Supplementary information [file 41467_2022_35476_MOESM1_ESM.pdf]

Supplementary Information for

Achieving single nucleotide sensitivity in direct hybridization genome imaging

Yanbo Wang[1,10], Wayne T. Cottle[1,10], Haobo Wang[2], Momcilo Gavrilov[1], Roger S. Zou[3], Minh-Tam Pham[4-7], Srinivasan Yegnasubramanian[5-7], Scott Bailey[1,2] and Taekjip Ha[1,3,8,9*]

[1]Department of Biophysics and Biophysical Chemistry, Johns Hopkins University School of Medicine, Baltimore, MD 21205, USA.

[2]Bloomberg School of Public Health, Johns Hopkins University School of Medicine, Baltimore, Maryland 21205, USA.

[3]Department of Biomedical Engineering, Johns Hopkins University, Baltimore, MD 21205, USA.

[4]Department of Urology, James Buchanan Brady Urological Institute, Johns Hopkins University School of Medicine, Baltimore, Maryland 21205, USA.

[5]Department of Oncology, Johns Hopkins University School of Medicine, Baltimore, Maryland 21205, USA.

[6]Sidney Kimmel Comprehensive Cancer Center, Johns Hopkins University School of Medicine, Baltimore, Maryland 21205, USA.

[7]Cellular and Molecular Medicine Graduate Program, Johns Hopkins University School of Medicine, Baltimore, Maryland 21205, USA.

[8]Department of Biophysics, Johns Hopkins University, Baltimore, MD 21218, USA.

[9]Howard Hughes Medical Institute, Baltimore, MD 21205, USA.

[10]These authors contributed equally to this work.

[*]To whom correspondence should be addressed: Taekjip Ha (tjha@jhu.edu)

Supplementary Note 1

Ideally, if there is no cleavage by the eCas9 nickase RNP in Step 1, the input DNA for ddPCR should be an SSB DNA, and the ddPCR should generate FAM+/HEX+ droplets because the top strand is still intact (Fig. 2a). However, even when we used dCas9 instead of eCas9 nickase in Step 1 (Fig. 2a, “If no cleavage” flowchart), we observed 71% FAM+/HEX+ droplets and 29% FAM-/HEX+ droplets (Supplementary Fig. 3c), instead of 100% FAM+/HEX+ droplets. This is likely because PCR amplification of the F1/R1 amplicon failed in around 30% droplets even though an intact template DNA strand (i.e., the top DNA strand in Fig. 2a) is present in those droplets. This could also explain that, in the previous study of DSB-ddPCR, about 7% droplets are FAM-/HEX+ even though the input DNA was an uncleaved control DNA[1]. Therefore, a genomic DNA molecule that was not cleaved by the eCas9 nickase RNP in Step 1 would become an SSB DNA as input of the ddPCR reaction in Step 2, which could result in either a FAM-/HEX+ droplet or a FAM+/HEX+ droplet (Fig. 2a).

Supplementary Note 2

In Fig. 2, we performed SSB-ddPCR assays using eCas9 nickase in complex with either gMUC4-TwoMM, a guide RNA with two mismatches against a target in *MUC4* gene (Fig. 2b), or gMUC4-OneMM that carries one mismatch against the same *MUC4* site (Fig. 2c). To estimate the true percentage of genomic DNA cleaved by the eCas9 nickase RNP in Step 1 (Fig. 2a) from the SSB-ddPCR readouts (i.e., the apparent DSB percentage), we generated a standard curve of the SSB-ddPCR assays. The complex of dCas9 with the gMUC4-OneMM was applied to fixed and permeabilized HEK293FT cells, and then genomic DNA was harvested (Supplementary Fig. 4a, Step 1). Next, the harvested genomic DNA was split into two portions. One was treated with restriction enzyme (MseI) to generate “DSB DNA”, and the other was treated with Cas9 nickase which cleaves the bottom strand to generate “SSB DNA” (Supplementary Fig. 4a, Step 2). Finally, DSB DNA and SSB DNA were mixed at different ratios for ddPCR. The relationship between the percentage of apparent DSB percentage and the percentage of DSB DNA added into the ddPCR reaction is linear (Supplementary Fig. 4b, Pearson’s $r^2 > 0.99$).

In the SSB-ddPCR assay (Fig. 2a), the percentage of DNA cleaved by the eCas9 nickase RNP in Step 1 equals the percentage of DNA carrying DSB (i.e., DSB DNA) added to the ddPCR reaction after Step 2. Therefore, by using the standard curve, we can estimate the percentage of DNA cleaved by the eCas9 nickase RNP in Step 1. For example, when performing the SSB-ddPCR assay using eCas9 nickase with gMUC4-TwoMM (Fig. 2a), the apparent DSB percentage was 29.4% ± 1.5% (Fig. 2b and 2d). According to the standard curve (Supplementary Fig. 4b), adding 0% DSB DNA (i.e., adding 100% SSB DNA) into ddPCR reaction resulted in 31.2% ± 1.7% apparent DSB percentage, insignificantly different from the apparent DSB percentage of the gMUC4-TwoMM SSB-ddPCR results. However, adding 5% DSB DNA into ddPCR reaction resulted in 35.5% ± 2.6% apparent DSB percentage, significantly larger than the apparent DSB percentage of the gMUC4-TwoMM SSB-ddPCR results (Supplementary Fig. 4b). These data suggest that when performing SSB-ddPCR using eCas9 nickase with gMUC4-TwoMM, less than 5% DSB DNA was added into the ddPCR reaction (Supplementary Fig. 4b). Therefore, the DNA cleavage efficiency of eCas9 nickase in complex with gMUC4-TwoMM was less than 5% (Fig. 2d). When performing the SSB-ddPCR assay using eCas9 nickase with gMUC4-OneMM, the apparent DSB percentage was 58.0% ± 1.0% (Fig. 2c and 2d). According to the standard curve, adding 40% DSB DNA into ddPCR reaction resulted in 58.7% ± 6.3% apparent DSB percentage, insignificantly different from the apparent DSB percentage of the gMUC4-OneMM SSB-ddPCR

results (Supplementary Fig. 4c). These data suggest that when performing SSB-ddPCR using eCas9 nickase with gMUC4-OneMM, ~ 40% DSB DNA was added into the ddPCR reaction (Supplementary Fig. 4c). Therefore, the DNA cleavage efficiency of eCas9 nickase in complex with gMUC4-OneMM was around 40% (Fig. 2d).

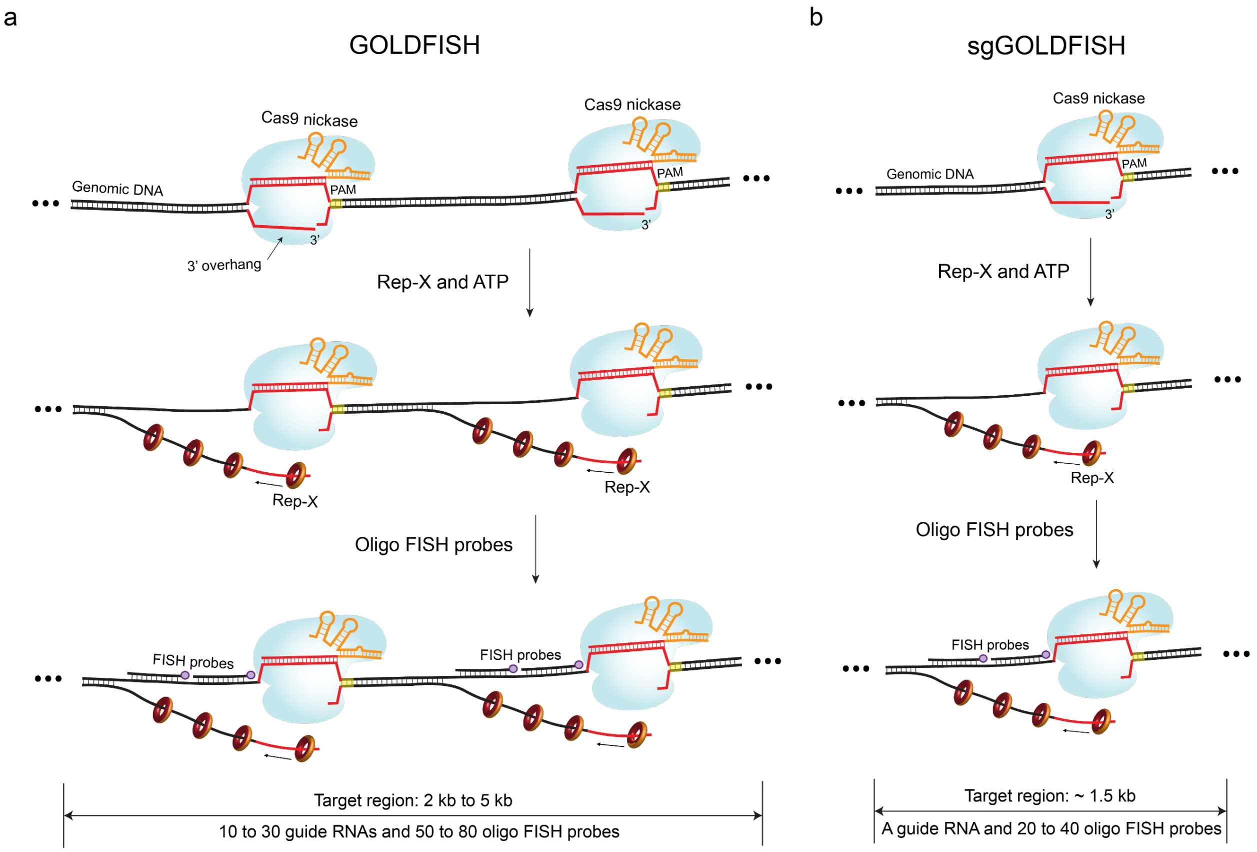

Supplementary Fig. 1|GOLDFISH and sgGOLDFISH. a, Schematic of GOLDFISH. Cas9 nickase RNP is applied to fixed and permeabilized cells to cleave the genomic DNA. Then Rep-X along with ATP is added to unwind the genomic DNA from the Cas9 cleavage sites. Finally, fluorescently labeled FISH probes are added to hybrid to sequences of interest. Multiple different guide RNA species and FISH probes are used in the GOLDFISH. The target region (i.e., guide RNA and probe binding sites) spans typically 2 kb to 5 kb. **b**, Schematic of sgGOLDFISH. The experimental procedure is the same as GOLDFISH, but only 1 guide RNA species is used in the sgGOLDFISH. The target region spans typically 1 kb to 1.5 kb.

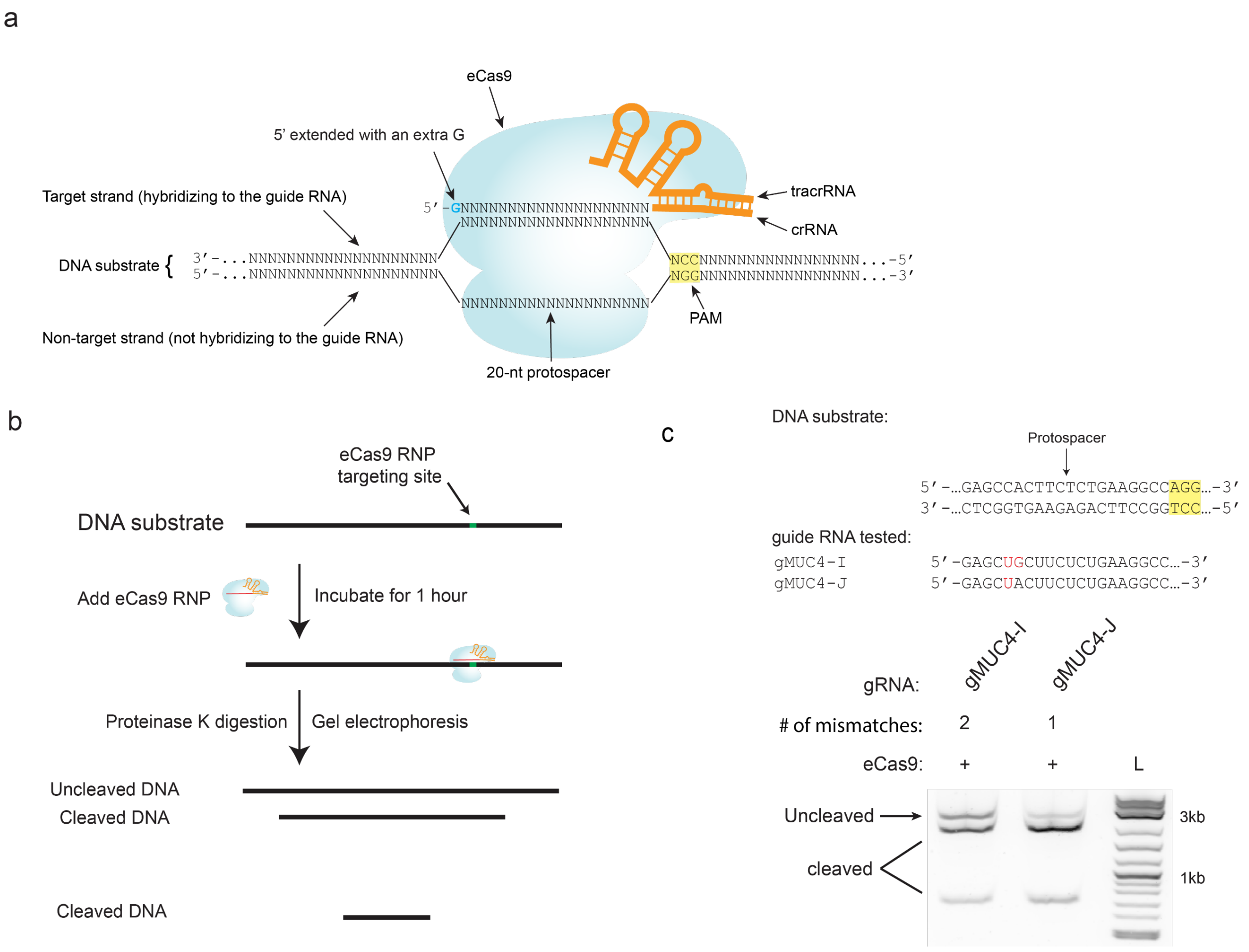

Supplementary Fig. 2 | eCas9 RNP and the *in vitro* cleavage assay. a, Schematic of eCas9 RNP. Compared to canonical guide RNA, the 5' extended guide RNA used in this study has an extra guanine (bolded in the figure) at the 5' of crRNA. **b**, Schematic of *in vitro* cleavage assay. eCas9 RNP was mixed with DNA substrate and incubated for 1 hour at 37 °C. Then proteinase K was added to digest bound and free eCas9. Finally, the reaction was loaded into an agarose gel for electrophoresis. **c**, Gel image of the *in vitro* cleavage assay using canonical guide RNA (i.e., without the 5' extended guanine). Significant cleavage activity was observed with the canonical guide RNA even if there are two mismatches between the guide RNA and DNA substrate. Each experiment was repeated independently twice with similar results. L, molecular weight markers.

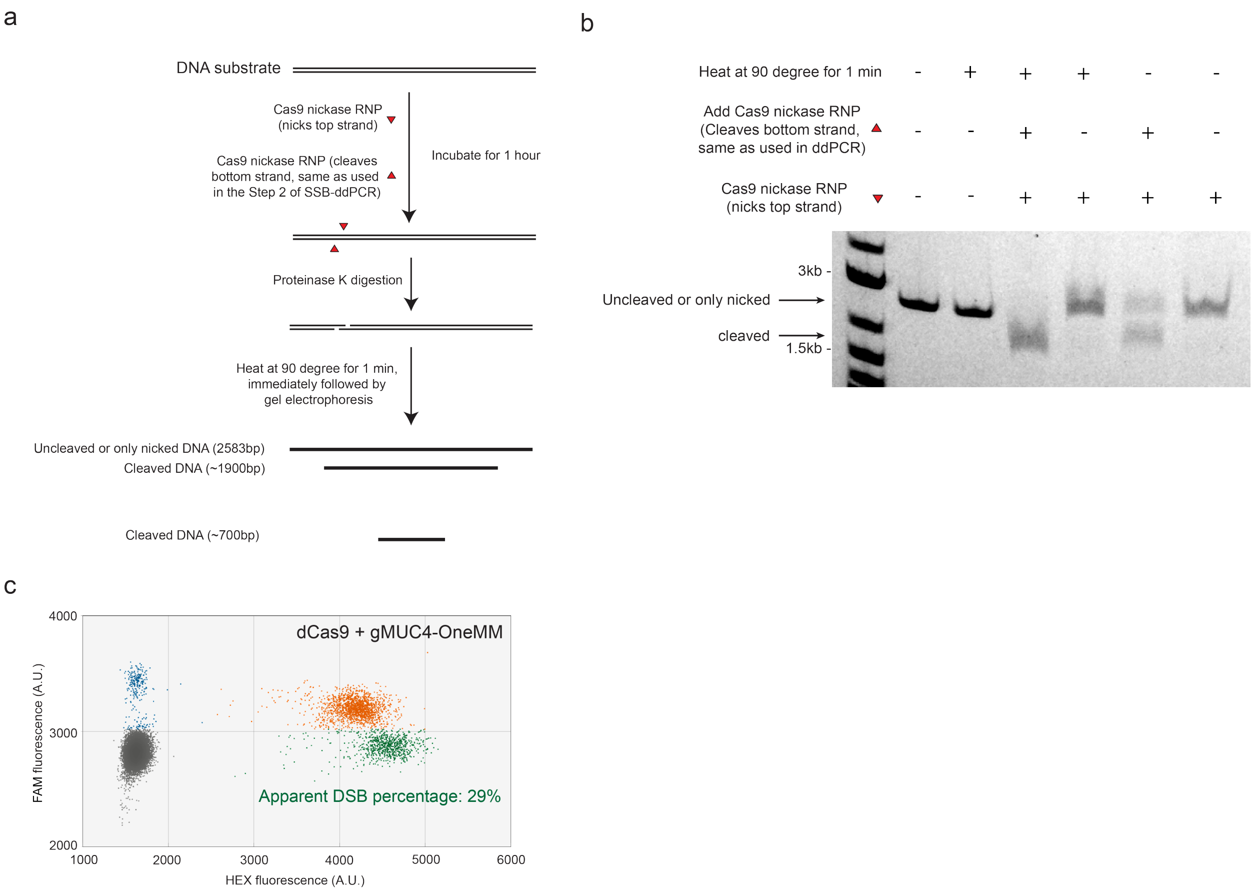

Supplementary Fig. 3|Control experiments for SSB-ddPCR. a, *In vitro* cleavage assay to measure the efficiency of DNA cleavage by Cas9 nickase RNP in the step 2 in Fig. 2a. In this assay, less than 8 ng/μL PCR-synthesized DNA substrate (containing the *MUC4* SSB-ddPCR targeting region) was mixed with 400 nM Cas9 nickase RNP cleaving the top strand and 400 nM Cas9 nickase RNP cleaving the bottom strand, and incubated for 1 hour at 37 °C. The 400 nM Cas9 RNP cleaving the bottom strand was also used in the Step 2 in Fig. 2a. After proteinase K treatment, the reaction was heated at 90 °C for 1 min to dissociate the two parts of the double-nicked DNA, followed by agarose gel electrophoresis. **b**, Gel image of the *in vitro* cleavage assay. Only the 3rd lane shows close to 100% cleavage efficiency indicates the 400 nM Cas9 RNP cleaved almost all DNA molecules. Each experiment was repeated independently twice with similar results. **c**, Representative SSB-ddPCR result using dCas9 and gMUC4-OneMM. Apparent DSB percentage is shown in green.

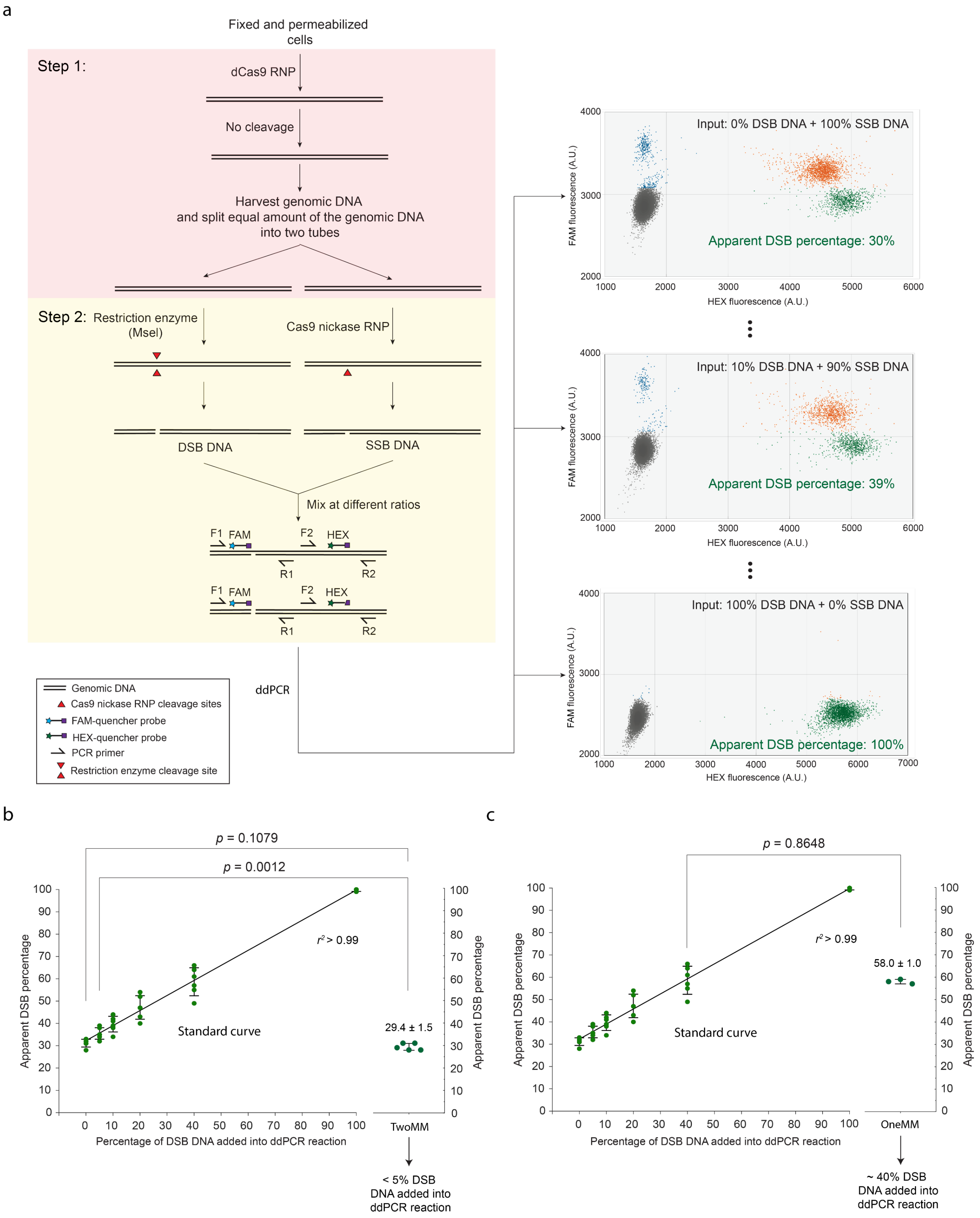

Supplementary Fig. 4 | Generating standard curve of SSB-ddPCR. a, Schematic of a control experiment to generate the standard curve of SSB-ddPCR. **b**, The standard curve generated from Supplementary Fig. 4a and the scatter plot of the SSB-ddPCR result (each dot represents measured apparent DSB percentage

from a replicate) from eCas9 nickase in complex with gMUC4-TwoMM (same as shown in Fig. 2d). Error bar represents average apparent DSB percentage ± standard deviation, which is also labeled above data points for TwoMM. According to the standard curve, adding 0% DSB DNA into ddPCR reaction resulted in 31.2% ± 1.7% apparent DSB percentage, insignificantly different from the apparent DSB percentage of the gMUC4-TwoMM SSB-ddPCR results (29.4% ± 1.5%). However, adding 5% DSB DNA into ddPCR reaction resulted in 35.5% ± 2.6% apparent DSB percentage, significantly larger than the apparent DSB percentage of the gMUC4-TwoMM SSB-ddPCR results. Therefore, when performing the SSB-ddPCR using eCas9 nickase with gMUC4-TwoMM, less than 5% DSB DNA was added into the ddPCR reaction, hence less than 5% genomic DNA was cleaved by eCas9 nickase with gMUC4-TwoMM in Step 1 in Fig. 2a. **c**, The standard curve generated from Supplementary Fig. 4a and the scatter plot of the SSB-ddPCR result (each dot represents measured apparent DSB percentage from a replicate) from eCas9 nickase in complex with gMUC4-OneMM (same as shown in Fig. 2d). Error bar represents average apparent DSB percentage ± standard deviation, which is also labeled above data points for OneMM. According to the standard curve, adding 40% DSB DNA into ddPCR reaction resulted in 58.7% ± 6.3% apparent DSB percentage, insignificantly different from the apparent DSB percentage of the gMUC4-OneMM SSB-ddPCR results (58.0% ± 1.0%). Therefore, when performing the SSB-ddPCR using eCas9 nickase with gMUC4-OneMM, ~ 40% DSB DNA was added into the ddPCR reaction, hence around 40% genomic DNA was cleaved by eCas9 nickase with gMUC4-OneMM in Step 1 in Fig. 2a. Student's t test (two-sided) is used. The exact *p* values are labeled. The standard curve was generated from n=6 independent ddPCR replicates. The scatter plot for TwoMM in Supplementary Fig. 4b was generated from n=5 independent ddPCR replicates. The scatter plot for OneMM in Supplementary Fig. 4c was generated from n=3 independent ddPCR replicates. See Supplementary Note 2 for detailed descriptions. Raw data points underlying each plot are provided as a Source Data file.

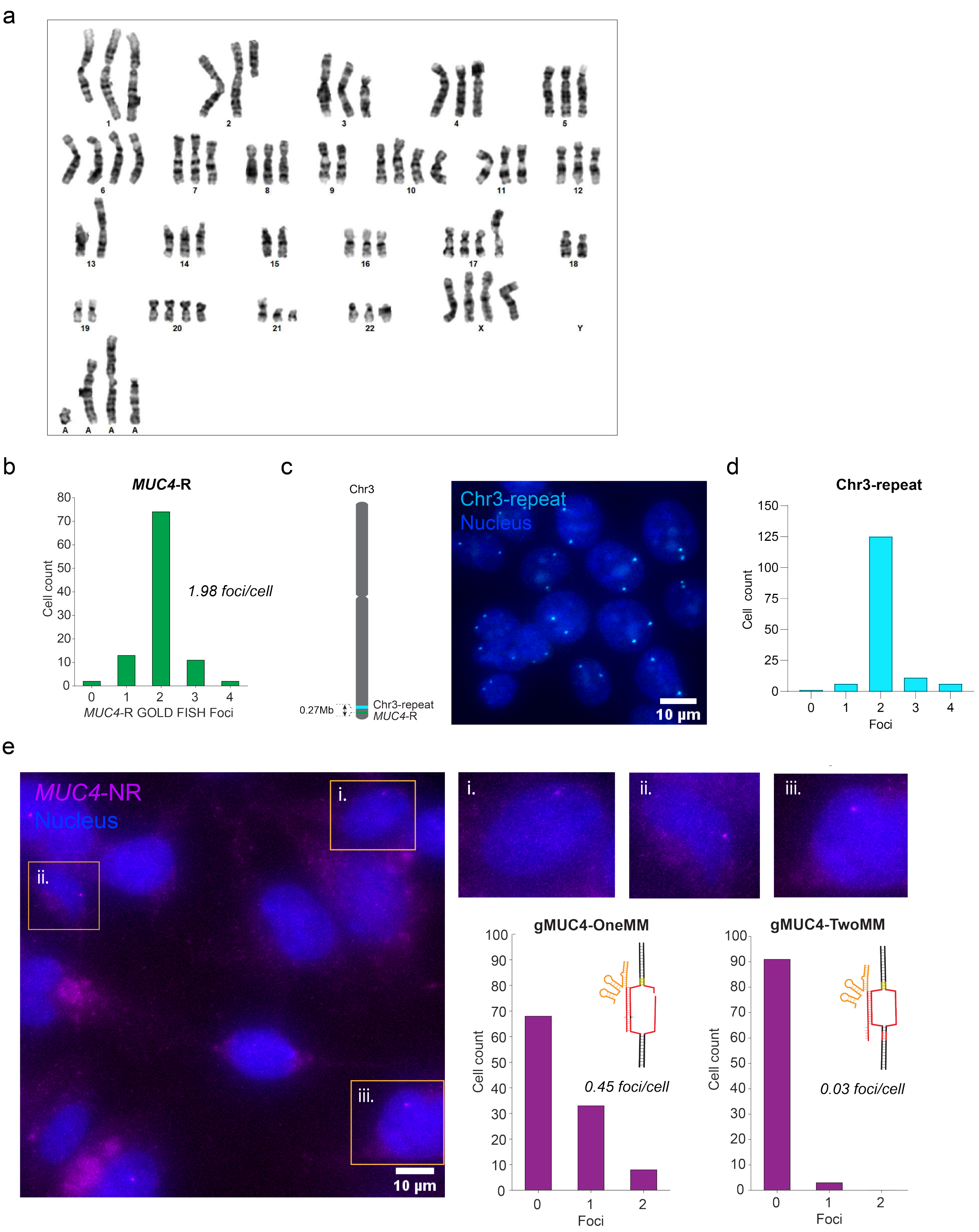

Supplementary Fig. 5 | Schematic of sgGOLDFISH against the *MUC4*-NR region and GOLDFISH against the *MUC4*-R region. a, Karyotyping of the HEK293 FT cell line used in Fig. 3. **b**, Histogram of *MUC4*-R GOLDFISH foci. **c**, Left, Schematic of MUC4-R region and Chr3-repeat region on Chromosome 3. Right, a

representative image of CASFISH against Chr3-repeat region. **d**, Histogram of Chr3-repeat CASFISH foci (n=149). **e**, A representative sgGOLDFISH image using gMUC4-OneMM without proteinase treatment in HEK293FT cells (single cells outlined in orange are magnified on the upper–right corner, each cell has one detected *MUC4*-NR allele), and histograms of sgGOLDFISH foci using gMUC4-OneMM (n=109) or gMUC4-TwoMM (n=94). Raw data points underlying each plot are provided as a Source Data file.

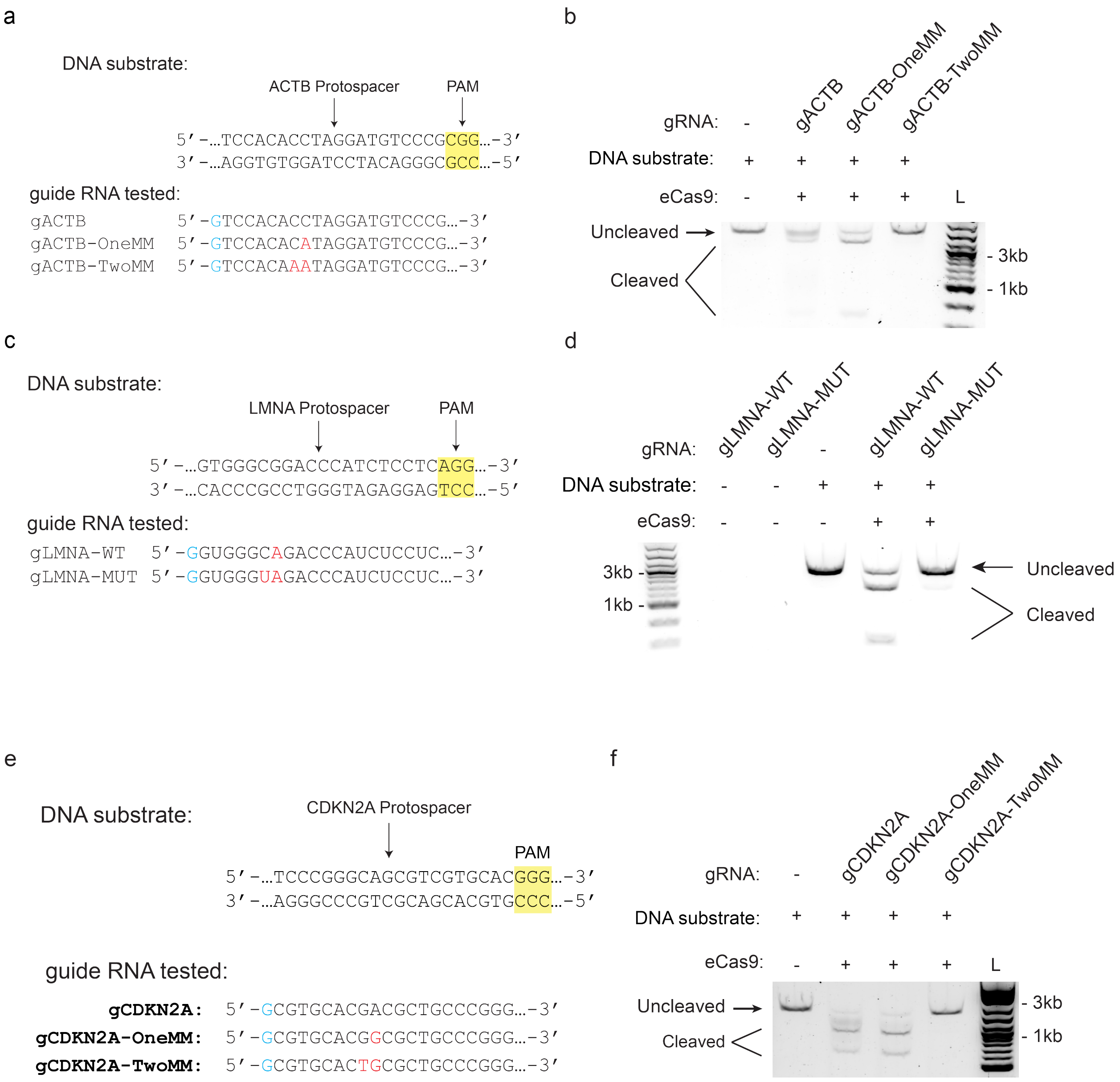

Supplementary Fig. 6|*In vitro* cleavage assay to measure cleavage activity of eCas9 in complex with gLMNA-MUT and gLMNA-WT against the *LMNA* gene. **a**, The DNA substrate was PCR-synthesized using human genomic DNA and primers against the *ACTB* gene. The sequences of 5′ end of gACTB, gACTB-OneMM and gACTB-TwoMM are shown. **b**, Gel image of the *in vitro* cleavage assay using guide RNAs against *ACTB.* **c**, The DNA substrate was PCR-synthesized using human genomic DNA and primers against the *LMNA* gene. The sequences of 5′ end of gLMNA-MUT and gLMNA-WT are shown. **d**, Gel image of the *in vitro* cleavage assay using guide RNAs against *LMNA*. **e**, The DNA substrate was PCR-synthesized using human genomic DNA and primers against the *CDKN2A* gene. The sequences of 5′ end of gCDKN2A, gCDKN2A-OneMM and gCDKN2A-TwoMM are shown. **f**, Gel image of the *in vitro* cleavage assay using guide RNAs against *CDKN2A*. The blue "G" represents the 5′ extended guanine of the crRNA. The red colored nucleotides represent mismatches against the DNA substrate. Each DNA cleavage experiment was repeated independently twice with similar results. L, molecular weight markers.

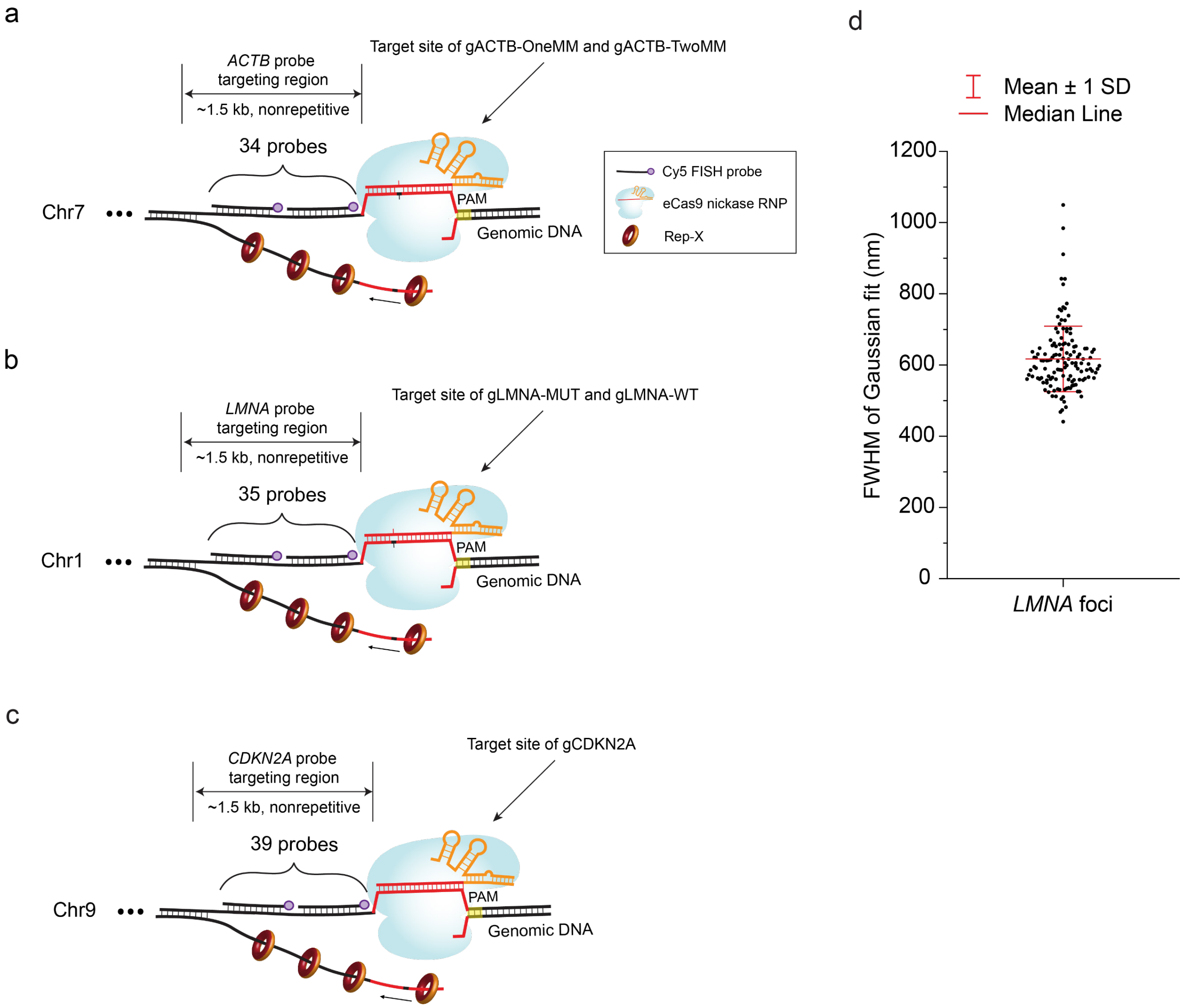

Supplementary Fig. 7|Schematics of sgGOLDFISH against *ACTB* and *LMNA*. a, Schematic of sgGOLDFISH against *ACTB* using gACTB-OneMM or gACTB-TwoMM. The figure shows the scenario that gACTB-OneMM is used (there is one mismatch between guide RNA and target protospacer). **b**, Schematic of sgGOLDFISH against *LMNA* using gLMNA-MUT or gLMNA-WT. The figure shows the scenario that gLMNA-WT is used to target a wild-type *LMNA* allele (there is one mismatch between guide RNA and target protospacer). **c**, Schematic of sgGOLDFISH against *CDKN2A* using gCDKN2A. **d**, Statistics of the full width at half maximum (FWHM) of the *LMNA* foci from the sgGOLDFISH using gLMNA-WT in Fig. 3e. Each dot represents a quantified *LMNA* focus (n=137). Median line is shown. Whisker represents mean ± standard deviation (SD). Raw data points underlying each plot are provided as a Source Data file.

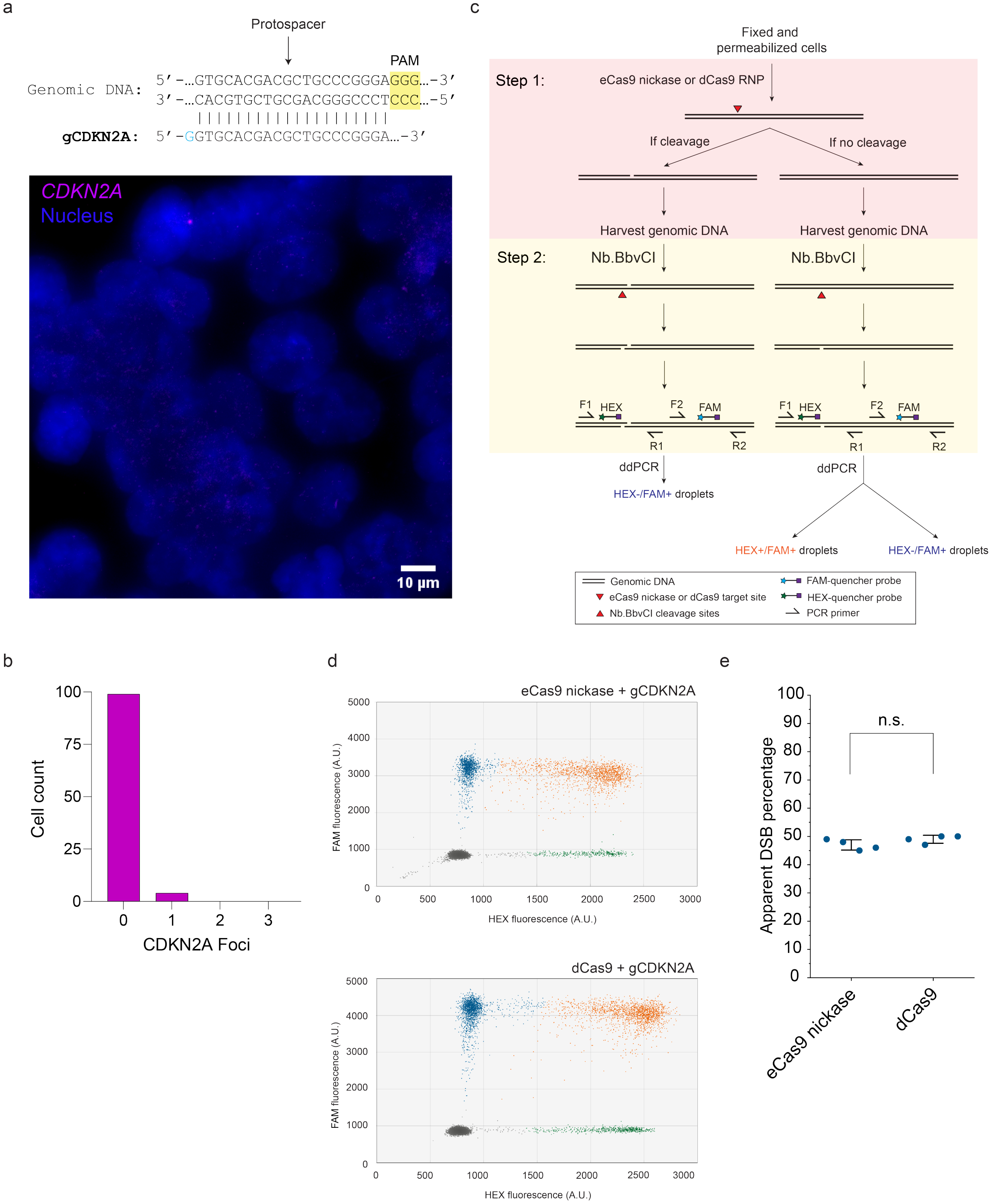

Supplementary Fig. 8|sgGOLDFISH against *CDKN2A*. **a**, Top, Sequences of *CDKN2A* target protospacer and gCDKN2A (only 21 nucleotides from the 5’ end of the crRNA are shown). The blue-colored G represents the extended guanine at the 5’ of the guide RNA. Experiment was repeated independently twice with similar results. **b**, Histogram of sgGOLDFISH foci using gCDKN2A. **c**, Schematic of *CDKN2A* SSB-ddPCR. **d**, Top, a representative *CDKN2A* SSB-ddPCR scattering plot. Bottom, a representative *CDKN2A*

SSB-ddPCR scattering plot using dCas9 instead of eCas9 nickase in the Step 1 of the SSB-ddPCR assay (Supplementary Fig. 8c). **e**, Scatter plot of percentages of apparent DSB percentage from the *CDKN2A* SSB-ddPCR using eCas9 nickase or dCas9. Each dot represents measured apparent DSB percentage from a replicate. Student's t test (two-sided) is used. n.s. represents $p > 0.05$. Error bar represents mean ± standard deviation from 4 replicates. Raw data points underlying each plot are provided as a Source Data file.

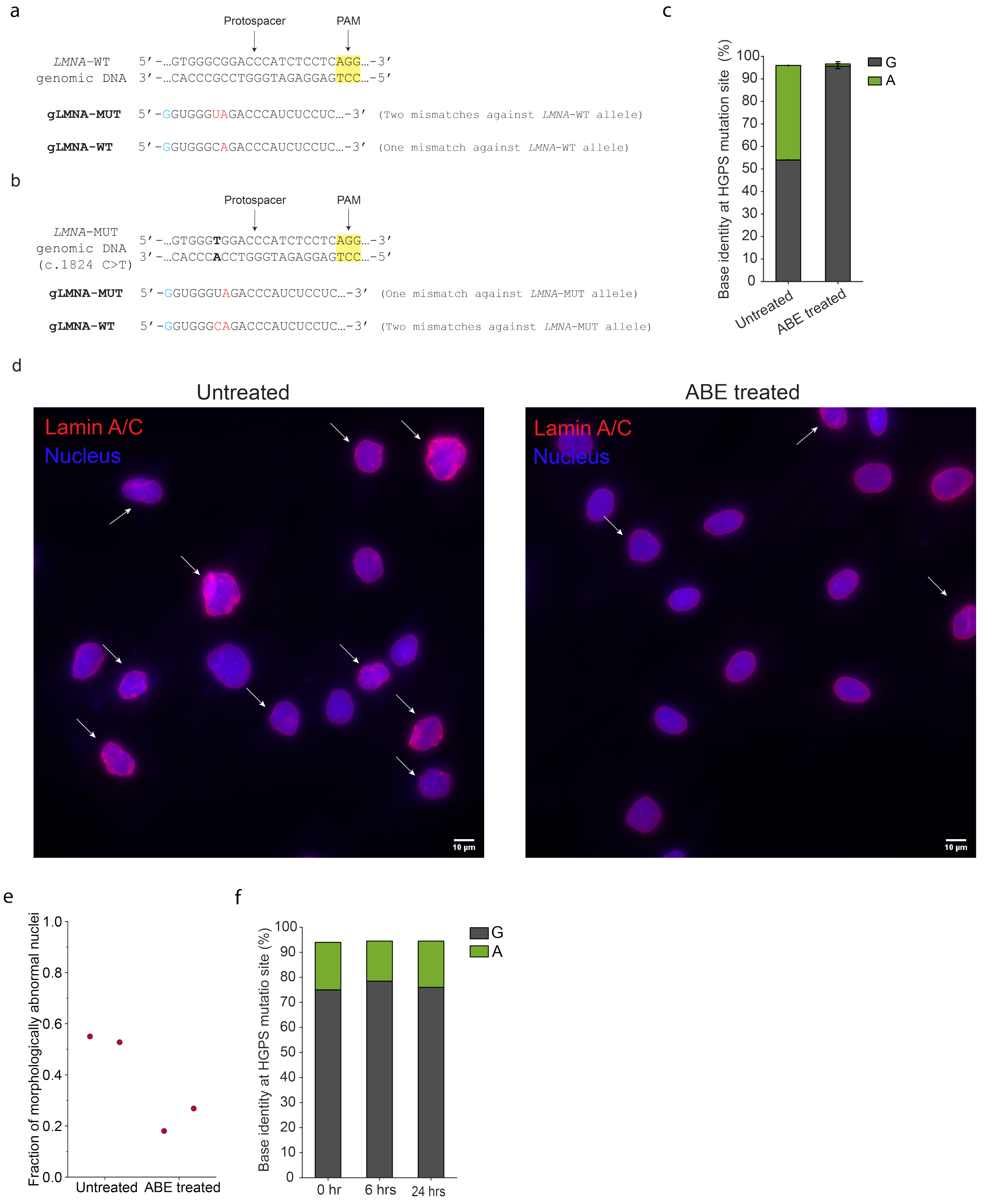

Supplementary Fig. 9|DNA-free base editing to correct the HGPS pathogenic point mutation. a, Sequences of the target protospacer of the *LMNA*-WT allele in HGPS cells and gLMNA-MUT or gLMNA-WT. The blue "G" represents the 5′ extended guanine of the guide RNA. The red colored nucleotides represent mismatches against the protospacer. **b,** Sequences of the target protospacer of the *LMNA*-MUT allele in

HGPS cells and gLMNA-MUT or gLMNA-WT. The blue “G” represents the 5’ extended guanine of the guide RNA. The red colored nucleotides represent mismatches against the protospacer. The bolded ‘A-T’ base pair indicates the *LMNA* c.1824 C>T mutation in HGPS fibroblasts. **c**, Base identity at the HGPS mutation site before and after ABE treatment. The error bar represents mean ± SD (n=3). **d**, Representative images showing the morphology difference of Lamin A/C meshwork between untreated and ABE-treated HGPS fibroblasts. White arrows indicate morphologically abnormal nuclei. **e**, Quantification of fraction of morphologically abnormal nuclei in untreated and ABE-treated HGPS fibroblasts. Morphologically abnormal nuclei were identified by visual inspection. The dataset was quantified twice by two persons independently. Each dot represents the quantified result from one person. More than 150 cells were quantified for each condition. **f**, Quantifications of base identity at the HGPS pathogenic point mutation site at different time points after mixing untreated and ABE-treated HGPS fibroblasts at 1:1 ratio (i.e., 1:1 mixture). Base identity was measured by Sanger sequencing. The data indicate the 1:1 mixture contains roughly 50% uncorrected and 50% ABE-corrected HGPS fibroblast within 24 hours. Raw data points underlying each plot and Sanger sequencing traces are provided as in Source Data files.

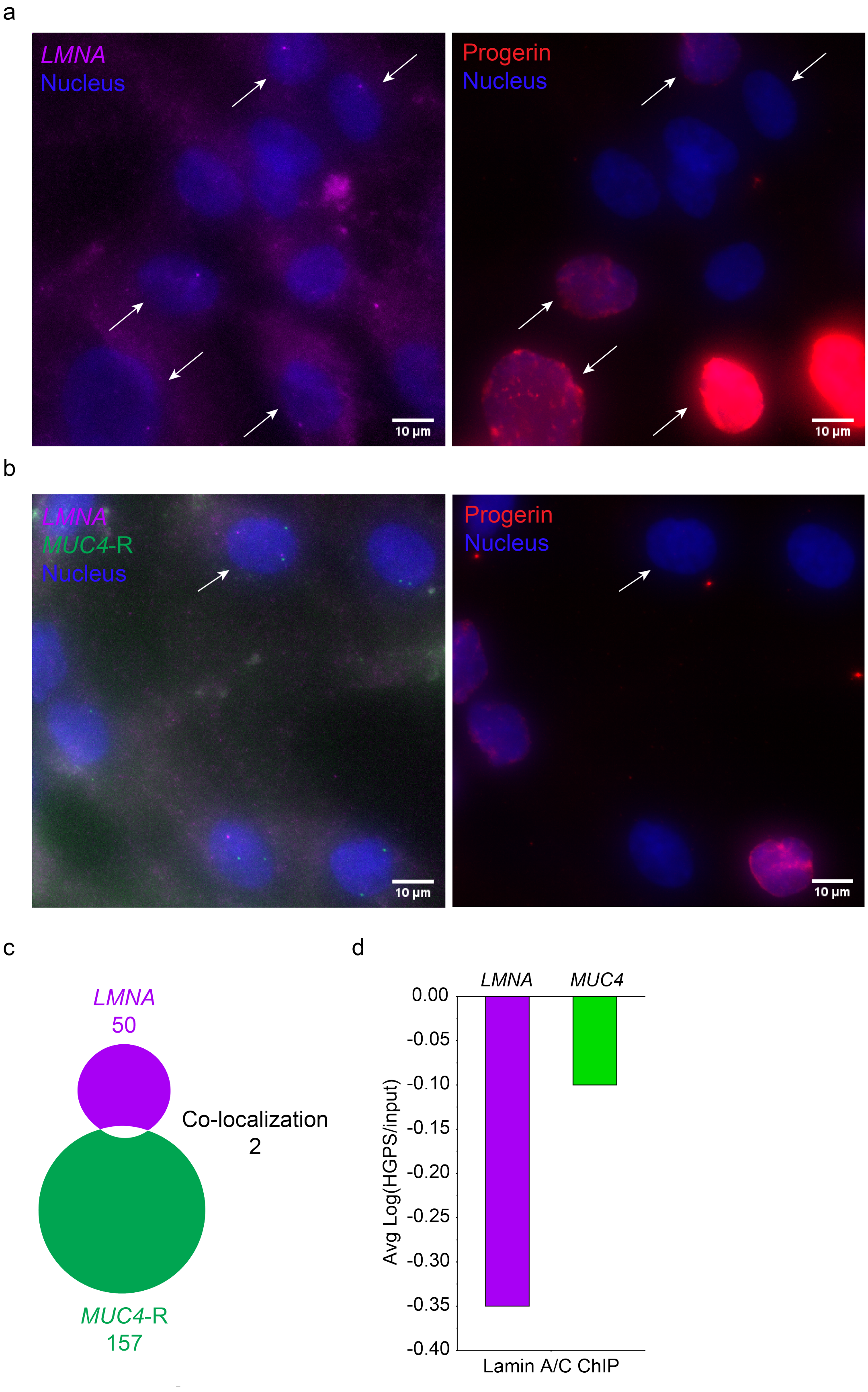
a
LMNA
Nucleus
10 µm
Progerin
Nucleus
10 µm
b
LMNA
MUC4-R
Nucleus
10 µm
Progerin
Nucleus
10 µm
c
LMNA
50
Co-localization
2
MUC4-R
157
d
LMNA
MUC4
0.00
-0.05
-0.10
-0.15
-0.20
-0.25
-0.30
-0.35
-0.40
Avg Log(HGPS/input)
Lamin A/C ChIP

Supplementary Fig. 10|sgGOLDFISH signals can be used for spatial analysis. a, A representative image of sgGOLDFISH against *LMNA* using gLMNA-MUT and progerin immunofluorescence in 1:1 mixture. The "mutant-positive cells" are indicated by white arrows. Experiment was repeated twice independently with similar results. **b**, A representative image of sgGOLDFISH against *LMNA* using gLMNA-WT and progerin immunofluorescence in 1:1 mixture. The "correction-positive cell" is indicated by a white arrow. Experiment was repeated twice independently with similar results. **c**, Quantification of co-localized *MUC4*-R and *LMNA* foci. **d**, Lamin A/C-ChIP data of the HGPS fibroblasts from a previous study (https://research.nhgri.nih.gov/manuscripts/Collins/HGPSepigenetics/). The enrichment (i.e., Avg Log(HGPS/Input)) of the *LMNA* and *MUC4* genes in Lamin A/C ChIP is shown. Smaller value (i.e., more negative) suggests less enriched in Lamin A/C ChIP. The data show that the *LMNA* gene is less enriched than the *MUC4* gene in Lamin A/C ChIP. This is consistent with our data in Fig. 4i showing that the *MUC4* alleles are closer to the nuclear edge than the *LMNA*-WT and *LMNA*-MUT alleles. Raw data points underlying each plot are provided as a Source Data file.

Supplementary Table 1| Comparison of sgGOLDFISH with other nuclear FISH methods. CasPLA relies on Cas9's binding specificity to discriminate SNVs, therefore limits the target SNVs within a protospacer and proximal to (< 10 bp) the protospacer adjacent motif (PAM)[2]. sgGOLDFISH relies eCas9 nickase's cleavage specificity to discriminate SNVs, hence allows for targeting SNVs distal to PAM. STAR-FISH is based on *in situ* PCR that produces cloud-like signals which reduces the localization accuracy of target SNVs[3], whereas in sgGOLDFISH probes directly hybridize to genome and produce well-defined signals. Furthermore, CasPLA and STAR-FISH requires proteinase treatment to detect nuclear SNVs[2, 3], while sgGOLDFISH does not require proteinase treatment. Zombie is limited to detect SNVs (e.g., SNVs generated by base editor) in pre-integrated DNA barcodes because it requires phage promoters upstream of the target SNV[4]. In contrast, there is no need to modify the genome of samples for sgGOLDFISH. Although amp-FISH was designed for detecting SNV in RNA[5], it has been implemented for gene locus identification by targeting the nascent RNA of expressing genes[6].

	**Nuclear FISH methods**					
	sgGOLDFISH	CasPLA[2]	STAR-FISH[3]	Zombie[4]	amp-FISH[5]	Live cell Cas9 imaging[7]
**Targeting scope of nuclear SNV**	PAM-distal (Yes) PAM-proximal (Possible)	PAM-proximal	3' end of a PCR primer	Pre-integrated DNA barcodes with phage promoters	Actively expressing genes	Not SNV sensitive
**Labeling efficiency of non-repetitive targets**	Moderate	Moderate	High	High	High	Moderate
**Global DNA denaturation**	Not Required	Not Required	Required	Not Required	Not Required	Not Required
**Protein immunofluorescence compatible**	Yes	No	No	Yes	Yes	No
**Live cell imaging**	No	No	No	No	No	Yes
**Tissue imaging**	Possible	Yes	Yes	Yes	Yes	Possible

Supplementary information references

1. Rose JC*, et al.* Rapidly inducible Cas9 and DSB-ddPCR to probe editing kinetics. *Nature methods* **14**, 891-896 (2017).

2. Zhang K*, et al.* Direct Visualization of Single-Nucleotide Variation in mtDNA Using a CRISPR/Cas9-Mediated Proximity Ligation Assay. *J Am Chem Soc* **140**, 11293-11301 (2018).

3. Janiszewska M*, et al.* In situ single-cell analysis identifies heterogeneity for PIK3CA mutation and HER2 amplification in HER2-positive breast cancer. *Nature genetics* **47**, 1212-1219 (2015).

4. Askary A*, et al.* In situ readout of DNA barcodes and single base edits facilitated by in vitro transcription. *Nature biotechnology* **38**, 66-75 (2020).

5. Marras SAE, Bushkin Y, Tyagi S. High-fidelity amplified FISH for the detection and allelic discrimination of single mRNA molecules. *Proceedings of the National Academy of Sciences* **116**, 13921-13926 (2019).

6. Fraser LCR, Dikdan RJ, Dey S, Singh A, Tyagi S. Reduction in gene expression noise by targeted increase in accessibility at gene loci. *Proceedings of the National Academy of Sciences* **118**, e2018640118 (2021).

7. Chen B*, et al.* Dynamic imaging of genomic loci in living human cells by an optimized CRISPR/Cas system. *Cell* **155**, 1479-1491 (2013).
