# Supplementary material for: Achieving single nucleotide sensitivity in direct hybridization genome imaging
Source: Nat Commun. 2022 Dec 15;13:7776. doi: 10.1038/s41467-022-35476-y (PMC9755149; doi:10.1038/s41467-022-35476-y)
Supplement: Supplementary file 2 — Reporting Summary [file 41467_2022_35476_MOESM2_ESM.pdf]

nature portfolio

Corresponding author(s): Taekjip Ha
Last updated by author(s): Dec 1, 2022

Reporting Summary

Nature Portfolio wishes to improve the reproducibility of the work that we publish. This form provides structure for consistency and transparency in reporting. For further information on Nature Portfolio policies, see our Editorial Policies and the Editorial Policy Checklist.

Statistics

For all statistical analyses, confirm that the following items are present in the figure legend, table legend, main text, or Methods section.

n/a	Confirmed	
☐	☒	The exact sample size (n) for each experimental group/condition, given as a discrete number and unit of measurement
☐	☒	A statement on whether measurements were taken from distinct samples or whether the same sample was measured repeatedly
☐	☒	The statistical test(s) used AND whether they are one- or two-sided<br>*Only common tests should be described solely by name; describe more complex techniques in the Methods section.*
☒	☐	A description of all covariates tested
☒	☐	A description of any assumptions or corrections, such as tests of normality and adjustment for multiple comparisons
☐	☒	A full description of the statistical parameters including central tendency (e.g. means) or other basic estimates (e.g. regression coefficient) AND variation (e.g. standard deviation) or associated estimates of uncertainty (e.g. confidence intervals)
☐	☒	For null hypothesis testing, the test statistic (e.g. F, t, r) with confidence intervals, effect sizes, degrees of freedom and P value noted<br>*Give P values as exact values whenever suitable.*
☒	☐	For Bayesian analysis, information on the choice of priors and Markov chain Monte Carlo settings
☒	☐	For hierarchical and complex designs, identification of the appropriate level for tests and full reporting of outcomes
☐	☒	Estimates of effect sizes (e.g. Cohen's d, Pearson's r), indicating how they were calculated

Our web collection on statistics for biologists contains articles on many of the points above.

Software and code

Policy information about availability of computer code

Data collection	NIS-Elements AR were used for collecting imaging data.
Data analysis	Fiji (ImageJ - 2.0.0-rc-61/1.51n, Java - 1.8.0_66) and Matlab (R2021b) were used for imaging data processing. OriginPro 2021b was used for generating plots. Oligoarray 2.1 was used to generate oligo probes. Nuclear edge detection was analyzed using custom code provided here: https://github.com/ywang285/NuclearEdge

For manuscripts utilizing custom algorithms or software that are central to the research but not yet described in published literature, software must be made available to editors and reviewers. We strongly encourage code deposition in a community repository (e.g. GitHub). See the Nature Portfolio guidelines for submitting code & software for further information.

Data

Policy information about availability of data

All manuscripts must include a data availability statement. This statement should provide the following information, where applicable:

- Accession codes, unique identifiers, or web links for publicly available datasets
- A description of any restrictions on data availability
- For clinical datasets or third party data, please ensure that the statement adheres to our policy

The data generated in this study (e.g., z-stack raw images) have been deposited in at Mendeley data (doi: 10.17632/mh9mzgz2nn.1). Source data are provided with this paper. The GRCh38.p13 Primary Assembly was used in this study and downloaded from NCBI (https://www.ncbi.nlm.nih.gov/assembly/GCF_000001405.39/). The Lamin A/C-ChIP data of the HGPS fibroblasts was downloaded from NHGRI (https://research.nhgri.nih.gov/manuscripts/Collins/HGPSepigenetics/

download.shtml).

Human research participants

Policy information about studies involving human research participants and Sex and Gender in Research.

Reporting on sex and gender	N/A
Population characteristics	N/A
Recruitment	N/A
Ethics oversight	N/A

Note that full information on the approval of the study protocol must also be provided in the manuscript.

Field-specific reporting

Please select the one below that is the best fit for your research. If you are not sure, read the appropriate sections before making your selection.

☒ Life sciences ☐ Behavioural & social sciences ☐ Ecological, evolutionary & environmental sciences

For a reference copy of the document with all sections, see nature.com/documents/nr-reporting-summary-flat.pdf

Life sciences study design

All studies must disclose on these points even when the disclosure is negative.

Sample size	No statistical method was used to predetermine sample size. We aimed at imaging many cells in different field of views (cell numbers imaged are indicated in figure legends), which we empirically found to be sufficient to obtain reproducible results.
Data exclusions	Cells with overlapped nuclei (cells are too close to each other) are excluded from quantification of FISH foci/cell.
Replication	Each experiment was repeated independently twice with similar results.
Randomization	The study did not involve the treatment of human subjects or laboratory animals, so the experiments were not randomized.
Blinding	No blinding was performed in this study because data analysis were conducted under the identical criteria, conditions, or parameters in each comparison.

Reporting for specific materials, systems and methods

We require information from authors about some types of materials, experimental systems and methods used in many studies. Here, indicate whether each material, system or method listed is relevant to your study. If you are not sure if a list item applies to your research, read the appropriate section before selecting a response.

Materials & experimental systems

n/a	Involved in the study	
☐	☒	Antibodies
☐	☒	Eukaryotic cell lines
☒	☐	Palaeontology and archaeology
☒	☐	Animals and other organisms
☒	☐	Clinical data
☒	☐	Dual use research of concern

Methods

n/a	Involved in the study	
☒	☐	ChIP-seq
☒	☐	Flow cytometry
☒	☐	MRI-based neuroimaging

Antibodies

Antibodies used	Progerin Monoclonal Antibody (13A4) (Thermo Scientific, 39966)<br>Goat anti-Mouse Secondary Antibody, Alexa Fluor 750 (Invitrogen, A-21037)<br>Anti-Lamin A + Lamin C antibody [4C11] (Abcam, ab238303)
Validation	All primary antibodies were validated by manufacturers:

Progerin Monoclonal Antibody (13A4) (Thermo Scientific, 39966), mouse, validated for western blot and immunocytochemistry: https://www.thermofisher.com/antibody/product/Progerin-Antibody-clone-13A4-Monoclonal/39965
Goat anti-Mouse Secondary Antibody, Alexa Fluor 750 (Invitrogen, A-21037), validated for western blot, flow cytometry, and immunofluorescence: https://www.thermofisher.com/antibody/product/Goat-anti-Mouse-IgG-H-L-Cross-Adsorbed-Secondary-Antibody-Polyclonal/A-21037
Anti-Lamin A + Lamin C antibody [4C11] (Abcam, ab238303) mouse monoclonal, knock-out validated: https://www.abcam.com/lamin-a--lamin-c-antibody-4c11-ab238303.html

Eukaryotic cell lines

Policy information about cell lines and Sex and Gender in Research

Cell line source(s)	HEK293FT cells were purchased from the American Type Culture Collection; Hutchinson-Gilford Progeria Syndrome (HGPS) fibroblasts were purchased from the Progeria Research Foundation.
Authentication	No authentication was performed during the development of the project.
Mycoplasma contamination	No mycoplasma contamination test was performed during the development of the project.
Commonly misidentified lines (See ICLAC register)	No commonly misidentified line was used in this study.
