# Supplementary material for: Achieving single nucleotide sensitivity in direct hybridization genome imaging
Source: Nat Commun. 2022 Dec 15;13:7776. doi: 10.1038/s41467-022-35476-y (PMC9755149; doi:10.1038/s41467-022-35476-y)
Supplement: Supplementary file 4 — Description of Additional Supplementary Files [file 41467_2022_35476_MOESM4_ESM.pdf]

Title: Supplementary Data 1

Description: DNA and RNA sequences used in this study. The DNA sequences include the sequence of sgGOLDFISH probes, template DNA for transcription of crRNA or sgRNA, PCR or ddPCR primers, and ddPCR probes. The RNA sequences include the sequence of sgRNA for the *LMNA* base editing and crRNA for Chr3 CASFISH.
