# Supplementary figures and images for: Achieving single nucleotide sensitivity in direct hybridization genome imaging
Source: Nat Commun. 2022 Dec 15;13:7776. doi: 10.1038/s41467-022-35476-y (PMC9755149; doi:10.1038/s41467-022-35476-y)

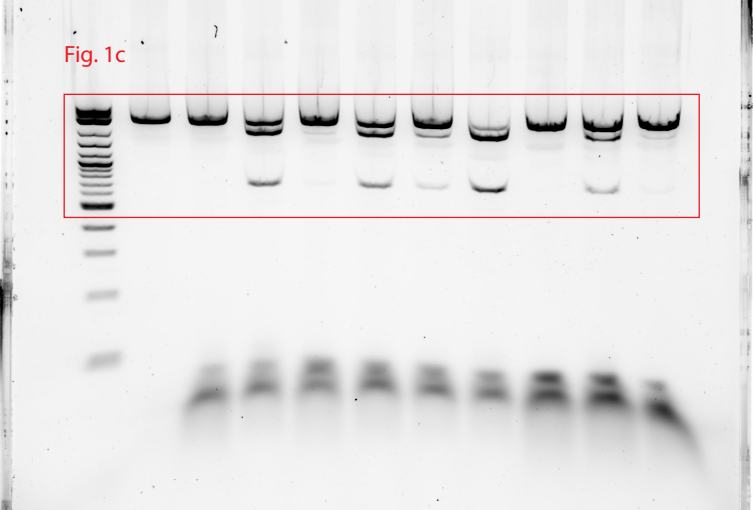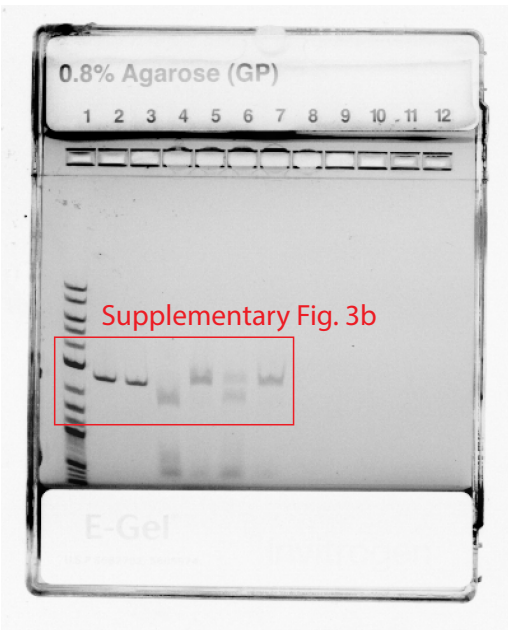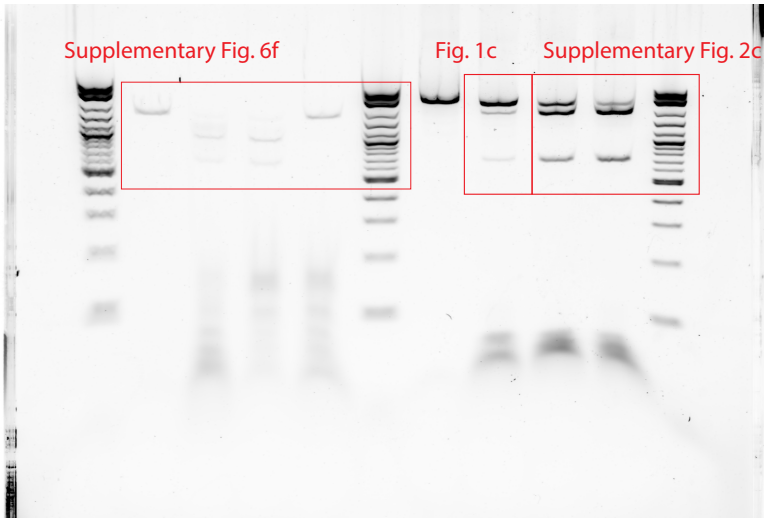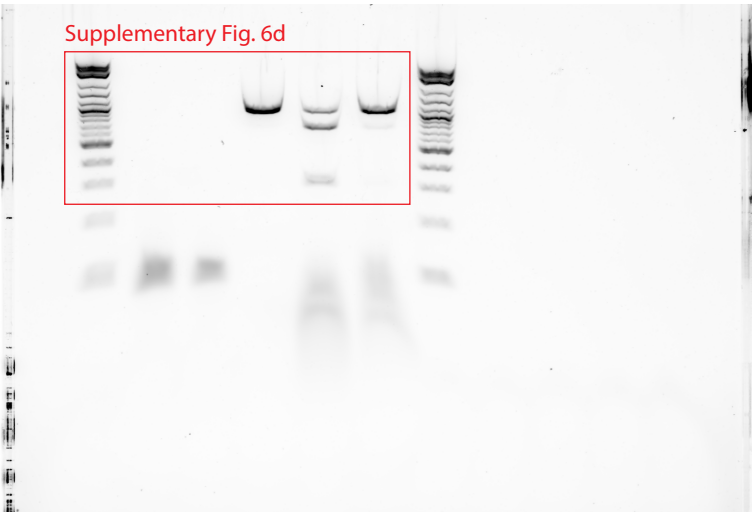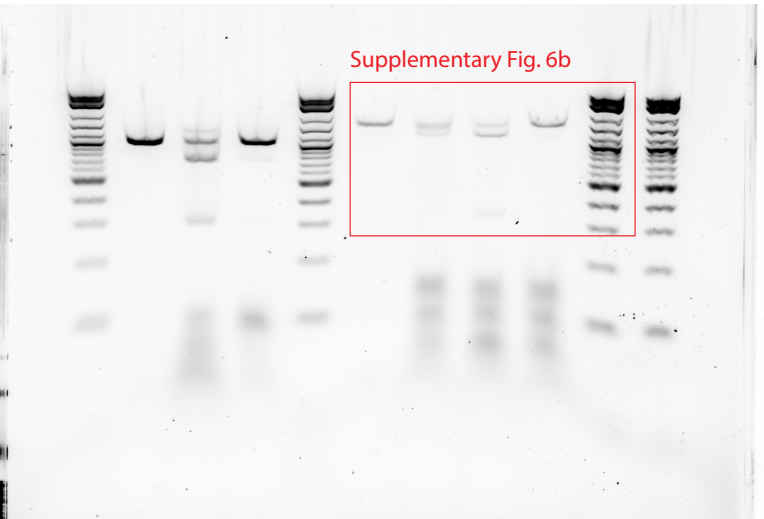

Supplement: Supplementary file 6 — Source Data [file 41467_2022_35476_MOESM6_ESM.zip › Source Data/Uncropped gel.pdf]
